# Successful surgical management of a Shamblin 3 carotid body tumor in Ghana: a case report

**DOI:** 10.11604/pamj.2026.53.124.47357

**Published:** 2026-03-11

**Authors:** Isaac Okyere, Solomon Obiri-Yeboah, Bryn Ansong, Samuel Gyasi Brenu

**Affiliations:** 1 Department of Surgery, School of Medical Sciences, Kwame Nkrumah University of Science and Technology, Kumasi, Ghana,; 2 Department of Oral and Maxillofacial Surgery, School of Dentistry, Kwame Nkrumah University of Science and Technology, Kumasi, Ghana,; 3 Directorate of Surgery, Komfo Anokye Teaching Hospital, Kumasi, Ghana

**Keywords:** Carotid body tumor, paraganglioma, Shamblin classification, surgery, case report

## Abstract

The carotid body is a parasympathetic paraganglion located at the carotid bifurcation. A carotid body tumor is a rare paraganglioma arising from the carotid body. They are benign tumors that often present as slow-growing, painless lateral neck masses. We present a 45-year-old woman with a 6-year history of a slowly progressive left lateral neck swelling, which was initially painless but subsequently became associated with intermittent, non-radiating, dull-aching pain. Examination showed a hemodynamically stable woman with an ovoid-shaped left lateral neck swelling extending from below the angle of the mandible to the supraclavicular region measuring 3cm x 5cm. A contrast-enhanced cervical computed tomography (CT) scan showed an intensely enhancing soft tissue within the left carotid sheath, which splays the internal and external carotid arteries and also encases the carotid bifurcation and internal jugular vein, making it a Shamblin 3 carotid body tumor, which is difficult to resect. She was worked up for neck exploration and excision biopsy under general anaesthesia. Post-operatively, there was no report of injuries to the spinal accessory, hypoglossal, or vagus nerves. She made an uneventful recovery and was discharged home. Complete surgical resection of carotid body tumors with no postoperative complications is feasible even in a low-resource setting.

## Introduction

Carotid bodies are positioned strategically at the bifurcation of the common carotid artery, which provides blood to the brain. These specialised structures or cells react to changes in oxygen, carbon dioxide, and metabolic acidosis, initiating quick respiratory responses to enhance oxygen supply and promote carbon dioxide removal. Additionally, carotid bodies can sense low glucose levels, temperature variations, and changes in osmolarity [1].

A carotid body tumor (CBT), also called carotid paraganglioma or a chemodectoma, is a rare type of neuroendocrine tumor [2]. Typically, benign CBT can develop due to prolonged hypoxia, such as from extended exposure to high altitudes. They are rare tumors and only appear in 1-2 individuals per 100,000 people [1]. CBT occurs frequently in adults, averaging 45-50 years of age and is uncommon in young age [3]. These tumors often present as asymptomatic, slow-growing, painless masses in the anterior neck. Early surgical resection is the gold standard for managing carotid body tumors (CBTs). This approach can be particularly challenging for Shamblin type III tumors, where the mass has grown to encase the carotid artery and surrounds the cranial nerves [4]. Due to the rarity of these cases, surgeons infrequently encounter them. Optimal management requires a thorough workup and a multidisciplinary approach. In this report, we present a case of CBT successfully treated with complete surgical resection in collaboration with the maxillofacial surgeon.

## Patient and observation

**Patient information:** a 45-year-old middle-aged woman with no known chronic illness presented with left neck swelling of 6 years' duration. The swelling gradually increased in size over the period and was associated with intermittent, non-radiating, dull ache pain with no alleviating or relieving factors, and there were no associated pressure symptoms.

**Clinical findings:** examination showed a hemodynamically stable woman with an ovoid-shaped left lateral neck swelling extending from below the angle of the mandible to the supraclavicular region measuring 3cm x 5cm. It was well-defined, non-tender, with no differential warmth, and exhibited mobility transversely but not vertically. There were no palpable cervical lymphadenopathy, and auscultation did not demonstrate any bruit.

**Diagnostic assessment:** a contrast-enhanced cervical CT scan showed an intensely enhancing soft tissue within the left carotid sheath, which splays the internal and external carotid arteries, producing the characteristic ''lyre'' appearance on sagittal reconstructed views. Complete encasement of the common carotid artery and internal jugular vein was noted at the C2-C4 vertebral levels. A magnetic resonance angiogram of the neck showed a T2-weighted hyperintense mass at the left carotid bifurcation measuring 3.4 x 2.6 x 4.3cm. Multiple flow voids were seen in the mass, causing displacement of the left external and internal carotid arteries.

**Therapeutic intervention:** she was worked up for neck exploration and excision biopsy under general anaesthesia via a cervical incision placed 2cm below the angle of the mandible. The hypoglossal nerve was identified and retracted medially. The encased internal jugular vein was ligated and divided. The common internal and external carotid arteries were identified and isolated. The vagus nerve was retracted posteriorly. The highly vascularized mass was carefully dissected off the bed from the proximal end to the carotid bifurcation with sequential hemostasis of branching arteries. An iatrogenic injury to the carotid bifurcation was repaired with polypropylene 4-0 while attempting to dissect the encasing mass off the common carotid artery. The incision was closed in layers over a vacuum drain.

**Follow-up and outcomes:** the patient made an uneventful postoperative recovery with no demonstrable neurological deficit to the vagus and hypoglossal nerves. She was discharged home after 3 days. Histopathologic analysis of the mass showed a circumscribed lesion with epithelioid cells arranged in clusters and nests and separated by fibrovascular stroma. The cells had uniform round nuclei and abundant granular amphophilic cytoplasm. Mitoses were rare, with no focal areas of necrosis.

**Patient perspective:** the patient was satisfied with the care and outcome of the intervention.

**Informed consent:** a signed informed consent was obtained.

## Discussion

**Figure 1:**
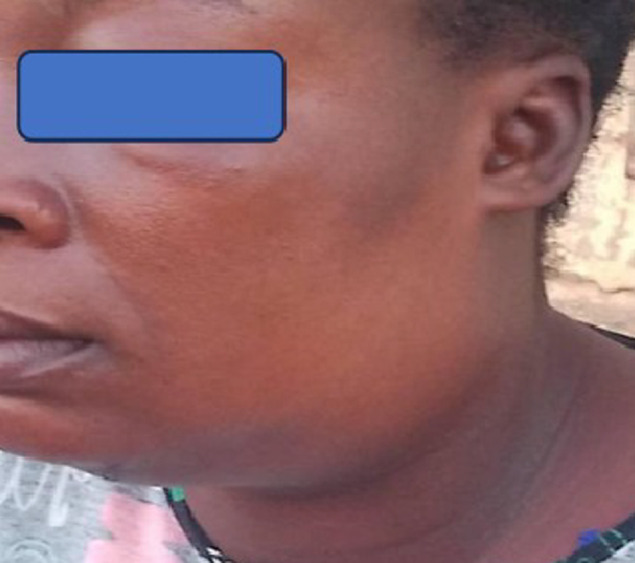
lesion in the left lateral neck

First described by Von Haller in 1743, the carotid body is a parasympathetic paraganglion located within the carotid sheath, appearing as a small, round, reddish-brown organ that is highly specialised and well defined, as shown in Figure 1. It is positioned in the adventitia of the carotid bifurcation. It receives its blood supply primarily from the ascending pharyngeal branch of the external carotid artery, with innervation from the vagus and glossopharyngeal nerves. Typically measuring 2-6 mm in diameter, the carotid body tends to be larger in individuals living at high altitude. Functionally, it acts as a chemoreceptor organ responding to hypoxia, hypercapnia, and acidosis, thereby regulating blood pressure, heart rate, respiration, and blood temperature through increased sympathetic activity [1,2].

Paragangliomas are tumors arising from sympathetic and parasympathetic paraganglia and most commonly occur in the head and neck region, where they present as CBTs. These tumors are typically well-defined, highly vascular, and rubbery-firm on palpation [3,4]. Three distinct forms of CBTs have been described: sporadic, hyperplastic, and familial. The sporadic type is the most common and typically presents between the ages of 45 and 50 years with no gender predilection [2]. The hyperplastic type is associated with chronic hypoxia and is commonly seen in patients with chronic obstructive pulmonary disease, cyanotic congenital heart disease, and residents of high-altitude regions. Familial CBTs occur at a younger age, are frequently associated with multiple head and neck paragangliomas, and carry a higher risk of malignancy. Approximately 10-15% of CBTs are familial, often following an autosomal dominant inheritance pattern with variable penetrance. Mutations in succinate dehydrogenase genes, particularly succinate dehydrogenase complex subunit D, succinate dehydrogenase complex iron sulfur subunit B, and succinate dehydrogenase complex subunit C, are strongly associated with familial disease, warranting genetic screening and counselling in affected families [1,3].

**Figure 2:**
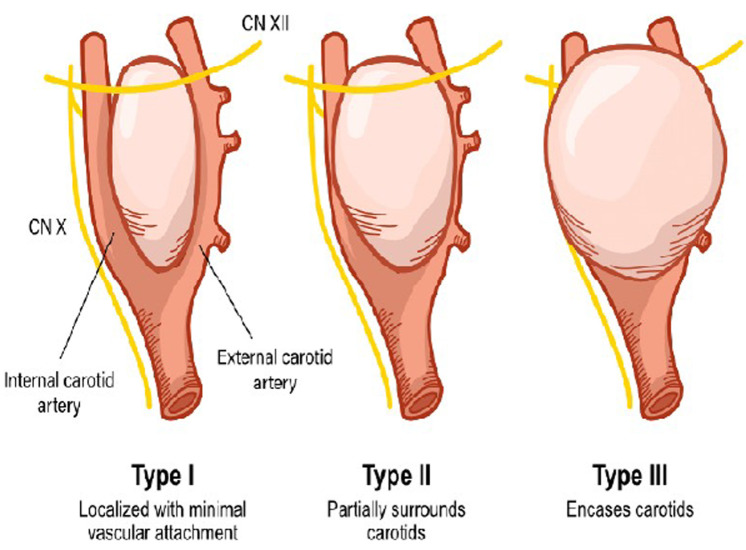
Shamblin classification of carotid body tumor

Shamblin classified carotid body tumors into three types based on their relationship with the carotid arteries (Figure 2). Type I tumors are small and easily dissected from the vessels, Type II tumors partially encase the carotid arteries and require meticulous dissection, while Type III tumors surround the carotid bifurcation and often necessitate vascular reconstruction [5]. A systematic review involving 4,418 patients with 4,743 CBTs demonstrated that approximately 44% were Shamblin Type II and 30% were Type III lesions [6].

**Figure 3:**
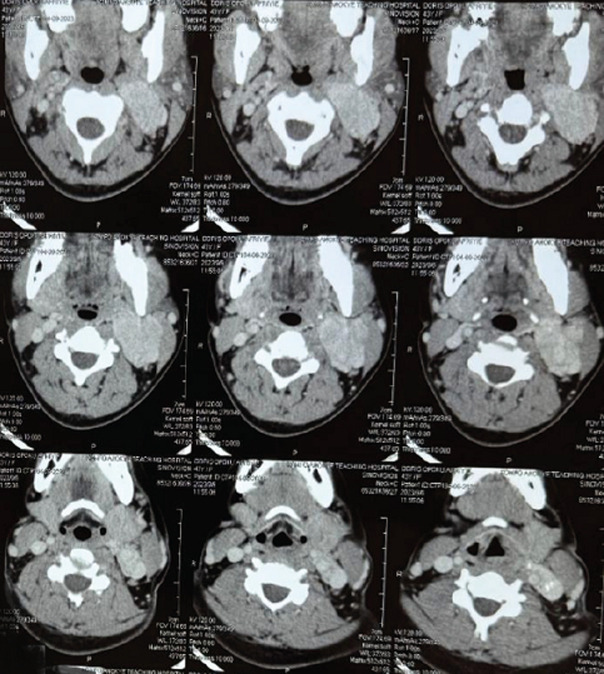
computed tomography angiography of the neck showing the carotid body tumor and its relationship with the internal and external carotid arteries

**Figure 4:**
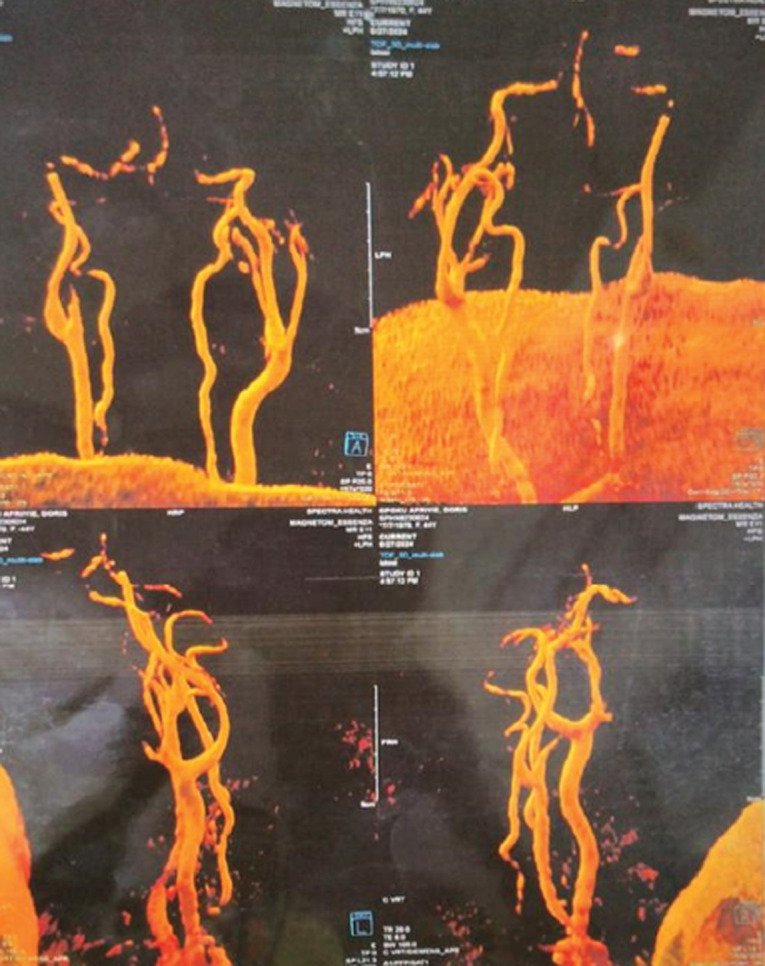
magnetic resonance angiography of the neck showing the carotid body tumor and its relationship with the internal and external carotid arteries

CBTs are often detected incidentally during physical examination or imaging. Diagnostic evaluation includes Doppler ultrasonography, CT, magnetic resonance angiography, and digital subtraction angiography (DSA). A CT scan was done for our patient, showing the mass (Figure 3). DSA remains the gold standard, characteristically demonstrating splaying of the internal and external carotid arteries (Lyre sign). However, colour Doppler ultrasonography provides a reliable, non-invasive alternative with high sensitivity and specificity [3,7]. Preoperative biopsy is contraindicated due to the risk of massive haemorrhage, pseudoaneurysm formation, tumor seeding, and carotid thrombosis [3] (Figure 4).

**Figure 5:**
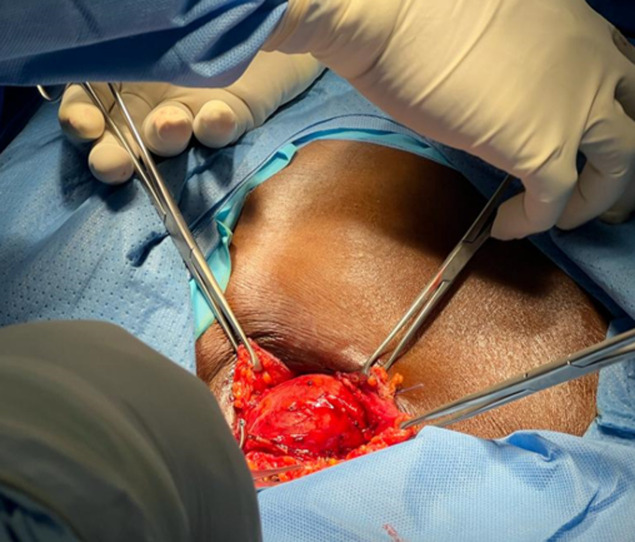
intraoperative picture of the carotid body

Complete surgical excision remains the treatment of choice for CBTs. Radiotherapy and embolisation are reserved for residual disease, unresectable tumors, or malignant cases with lymph node metastasis [5,8]. Despite the large size of the tumor in our patient, complete surgical excision was achieved (Figure 5), and histopathological analysis confirmed benign disease, eliminating the need for adjuvant radiotherapy. Surgical resection of Shamblin Type III tumors is technically demanding and associated with increased morbidity and mortality [9]. Tumors encasing the carotid bifurcation are particularly adherent and are best dissected last, following proximal and distal vascular control. Key surgical principles include early identification and preservation of cranial nerves, secure vascular control of the common and internal carotid arteries, and meticulous periadventitial dissection [5,9].

**Figure 6:**
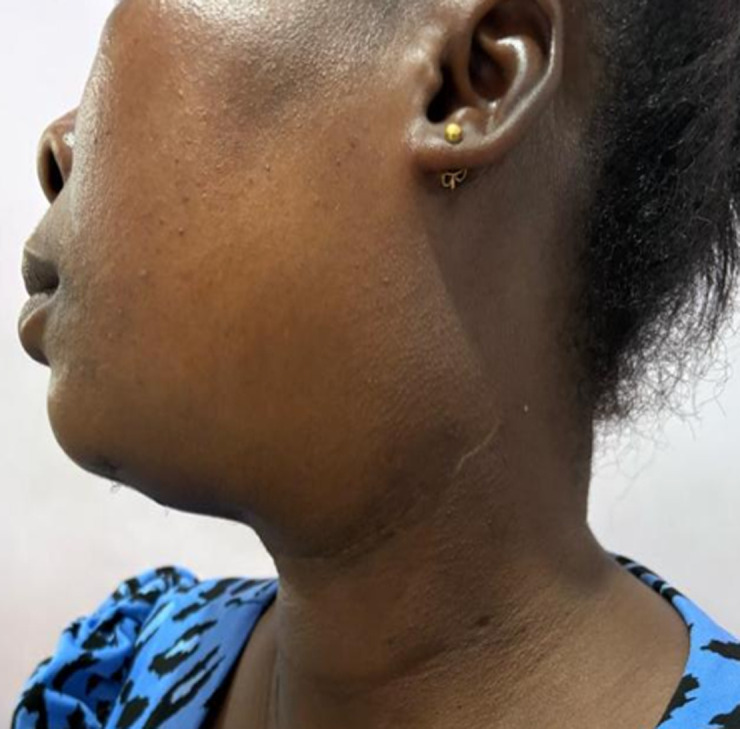
six months after surgery

Cranial nerve injury remains the most common complication following CBT resection, with hypoglossal nerve (cranial nerve XII) injury reported most frequently. Tumor proximity to the skull base and increasing tumor size are significant predictors of nerve injury [10]. Most nerve injuries resolve within six months, although permanent deficits have been reported [1,6]. In our case, no cranial nerve injury occurred. Postoperative care focuses on monitoring for neurological deficits, stroke, and hemodynamic instability, with regular follow-up imaging (Figure 6) to detect recurrence.

## Conclusion

A carotid body tumor is a rare paraganglioma arising from the carotid body. Although complete resection of the more complex Shamblin III carotid body tumor poses significant risks and challenges, especially in resource-limited centres, it is still achievable through careful preoperative evaluation and close collaboration with a maxillofacial surgeon.
